# Theoretically informed codesign of a tailored intervention to support pressure ulcer prevention behaviours by older people living in their own homes in the UK and their lay carers: an intervention codesign study (C-PrUP)

**DOI:** 10.1136/bmjopen-2023-083495

**Published:** 2024-11-07

**Authors:** Marjolein Woodhouse, Fiona Cowdell, Jennifer Roddis, Anne Devrell, Karen Oakley, Judith Dyson

**Affiliations:** 1 School of Health and Care Professions, University of Portsmouth, Portsmouth, UK; 2 Faculty of Health Education and Life Sciences, Birmingham City University, Birmingham, UK; 3 Patient and Public Involvement Representative, Birmingham, UK; 4 Adult Services, Solent NHS Trust, Portsmouth, UK; 5 C-SCHaRR, Birmingham City University, Birmingham, UK

**Keywords:** Nursing Care, Patient-Centered Care, Patient Participation, Primary Health Care, QUALITATIVE RESEARCH, Self-Management

## Abstract

**Objective:**

To codesign a theoretically underpinned, healthcare practitioner-mediated, tailored intervention to support housebound older patients and their lay carers to adopt pressure ulcer prevention behaviours.

**Design:**

Theoretical domains framework informed codesign.

**Setting:**

One geographical area in the UK, spanning several community National Health Service Trusts.

**Participants:**

Community-dwelling older patients at risk of pressure ulcer development and their lay carers (n=4) and health practitioners (n=6) providing related care.

**Results:**

Codesigners addressed five identified barriers to pressure ulcer prevention, knowledge and beliefs about consequences, social or professional role and influence, motivation and priorities, emotion and environment. Prioritised intervention components were (1) making every contact count, all health and social care workers to be conversant with basic prevention behaviours and to support and reiterate these at every visit (9.1/10), (2) signposting of existing support groups and sitting services (8.4/10), (3) accessible, timely, trustable and relatable written information including the role of patients, carers and staff in prevention and links to other resources (7.7/10) and (4) supporting close family involvement in some of the practical elements of care (5.6/10).

**Conclusions:**

Our study sought to codesign a practitioner-mediated, tailored intervention to support housebound older patients and their lay carers to adopt pressure ulcer prevention behaviours. The process of barrier identification and selection of behaviour change techniques for intervention components was theoretically informed. However, further development will be needed to refine the prototype intervention to take into account the complexity of multiple health needs and priorities of patients. The principles of this study are likely to be transferable to similar national and international contexts.

**STRENGTHS AND LIMITATIONS OF THIS STUDY**

- This is one of few studies to address pressure ulcer prevention among community-dwelling older adults.
- A structured theoretical approach was used to select the behaviour change techniques most likely to address identified barriers to pressure ulcer prevention.
- Patients, lay carers and healthcare practitioners engaged in codesigning the prototype intervention.
- Codesign processes had to be adapted to ensure inclusivity; therefore, direct dialogue between participants was limited.
- Recruitment was limited to a single geographical area, which may restrict transferability to other national and international contexts where needs and service provision could differ.

##  Introduction

Globally, pressure ulcers (PUs, also known as pressure injuries or informally as bedsores) are a common occurrence^1 2^ and place a substantial burden on health and social care services.^3^ PUs have an impact on quality of life^4^ and are associated with increased morbidity and mortality, pain, fear and despondency.^5 6^ International guidelines recommend preventative behaviours: (1) repositioning, (2) appropriate use of pressure-relieving devices (eg, pressure-relieving mattresses), (3) regular skin inspection and skin care and (4) optimal dietary and fluid intake.^7 8^ However, these guidelines focus on acute hospital populations and there is minimal research considering transferability to community settings.^9^ Implementing these guidelines in home care requires people at risk and lay carers to adopt certain actions and behaviours.

A James Lind Alliance PU Priority Setting Partnership^10^ identified the involvement of patients and lay carers in prevention as a key priority. Enabling at-risk community-dwelling people to engage in prevention behaviours has the potential to reduce PU incidence, cost of care and hospital admission rates. To date, most patient and lay carer PU prevention interventions have relied on the provision of information and these have had an uncertain impact on knowledge or PU development.^11^

We know that providing information alone is unlikely to change health behaviours and that explicit use of health behaviour theory increases likelihood of adoption of behaviours.^12 13^ The theoretical domains framework (TDF)^14^ is one such theory which synthesises all published theoretical constructs into 11 domains. These domains provide a detailed framework of determinants of behaviour (knowledge, skills, social/professional role and identity, beliefs about capabilities, beliefs about consequences, motivation and goals, memory attention and decision processes, environmental context and resources, social influences, emotion and action planning). When key determinants of specific behaviours have been identified, the TDF allows the identification of behaviour change techniques (BCTs) most likely to be effective.^15^ Unlike models of health behaviour or behaviour change, the TDF is accessible to and has been used with practitioners^16^ and service users^17^ and, therefore, supports the ‘working with’ principles of codesign. Interventions tailored to identified barriers and facilitators are more effective than untailored approaches.^18^ Our previous work identified barriers and facilitators to PU prevention behaviours in housebound older people and their lay carers.^19^

Codesigning interventions with end-users is known to increase uptake in practice,^20^ it can enhance innovation, improve performance and support equality, dignity and wellbeing.^21^ Effective codesign supports lay people and professionals working as equals at every stage in the research process.^22 23^ Codesigning behaviour change interventions is complex; to guide our approach we drew on (1) good practice guidance on coworking with older people,^24^ (2) the Medical Research Council guidelines for designing complex interventions,^25^ (3) our previous work qualitative work with patients, lay carers and healthcare practitioners (HCPs)^19^ and (4) a four-step systematic approach to designing theoretically informed interventions.^26^ We followed the first three steps of the latter as summarised in table 1.

**Table 1:** Process of intervention design

Step	Question	Action
1	Who needs to do what differently?	Using the (Action, Actor, Context, Target, Time) framework,^22^ we specified and defined desired PU prevention behaviours as older people at risk of pressure ulcers with the support of their lay (family) carers to engage in repositioning, skin care and inspection, use of pressure-relieving aids and best possible nutrition and hydration.
2	Using a theoretical framework, which barriers and facilitators need to be addressed?	We conducted TDF-based qualitative interviews to understand existing barriers and facilitators.^19^ We selected domains of importance according to the following criteria^41 42^ Frequency, the domain contained barriers for over half the participants. Personal importance, barriers expressed using emphatic language. Discordant views, domains that included both barriers and facilitators (suggesting barriers were modifiable).
3	Which intervention components (BCTs and mode(s) of delivery) could overcome the modifiable barriers and enhance the facilitators?	BCTs selected from a matrix where techniques are empirically mapped to the specific domains of the TDF^14^ and supplemented by additional BCTs taken from a taxonomy^43^ (based on the expertise of JD, CPsychol).

BCTsbehaviour change techniquesPUpressure ulcerTDFtheoretical domains framework

## Objective

To codesign a theoretically underpinned, HCP-mediated, tailored implementation intervention to support housebound older patients and their lay carers to adopt PU prevention behaviours.

## Methods

### Design

Codesign workshops to develop a prototype theoretically underpinned, HCP-mediated, tailored intervention to support housebound older patients and their lay carers to adopt PU prevention behaviours.

### Participants

We aimed to recruit up to seven HCPs, including a manager, and six patients or lay carers which we judged (based on similar precedent studies^27^,^29^) to be sufficient to develop a prototype, HCP-mediated, intervention to support patients and lay carers in PU prevention behaviours. It is estimated that in theoretically underpinned studies saturation is likely to be achieved with 15 participants.^30^ Participants included (1) patients, aged ≥65 years, living at home, receiving community healthcare and assessed as being at risk of developing a PU (irrespective of current and previous PU status), (2) lay carers providing unpaid care (for people fitting our patient criteria) of any kind (eg, physical, household or other practical support) and (3) HCPs providing care to patients as defined above. For patients, PU risk was determined by an HCP using local clinical protocols.

### Patient and public involvement

Two patient and public involvement colleagues were integral to this study. Both contributed to study design and one to the interpretation of data, reading and commenting on results and writing up. Staff at a local carers centre advised on and supported recruitment and hosted dissemination events.

### Recruitment

All patients and lay carers had been interviewed in our earlier study^19^ and indicated an interest in becoming codesigners. HCPs were recruited via partner care organisations by email invitation and word of mouth. Workshops took place between December 2022 and May 2023 and lasted between 47 min and 90 min. Participant information, including the reasons for the research, was provided, questions answered and written consent was given prior to the workshops. For those patients and lay carers who declined to take part, this was due to deterioration in health circumstances. All HCPs who requested participant information took part in at least one workshop. Participants were offered a voucher or payment in recognition of their contribution.

### Codesign workshops

**Figure 1:**
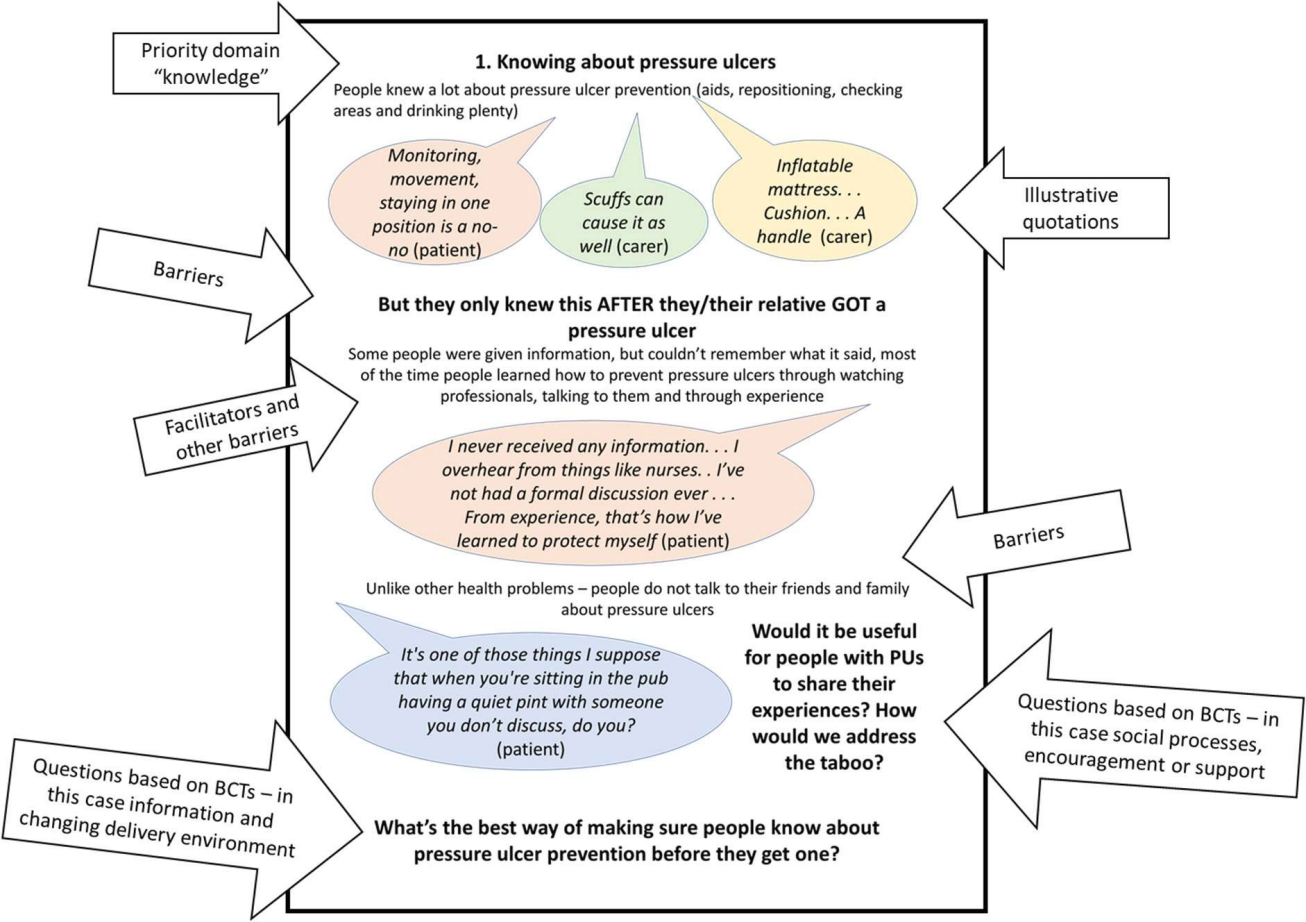
Example poster for discussion. BCTs, behaviour change techniques.

The six workshops involved a series of activities. The researchers presented posters (example in figure 1) identifying the barriers and facilitators to engaging in PU prevention behaviours, informed by the findings of and based around the barriers identified in our previous study^19^ (listed as themes in the results section below) and asked questions about how these might be addressed. We intended to run a series of codesign workshops involving both lay people and HCPs. However, health and mobility problems or carer responsibilities limited lay participation in group events. We, therefore, modified our approach, offering small group or one-to-one opportunities, in which we fed findings from previous workshops into subsequent ones. This modification was based on precedents from the literature and the experience of the research team.^27^,^29^ We conducted two group HCP workshops and individual sessions with one patient and three lay carers.

HCP workshops were conducted online using Microsoft Teams to facilitate maximum attendance and were held after usual working hours. The patient and lay carers all preferred a home visit from the research team at a convenient time. In some carer workshops, the person cared for was present during the workshop, though did not actively participate. Codesign sessions were facilitated by female, postdoctoral researchers (JR, MW and JD in combination). The researchers had no existing relationship with lay participants. Some HCP participants were known to one researcher (MW) in a professional capacity only. Where possible, sessions were held at least 2 weeks apart, however, where this was not possible due to participant availability, the researchers undertook a rapid analysis to synthesise data to feed back and forth, based on both data and fieldnotes.

Key considerations for codesigners included (1) the content of strategies (what was included), (2) mode of delivery (how strategies were delivered) and (3) actual or anticipated APEASE^31^ criteria (Acceptability, Practicability, Affordability, Side effects and Equity). The discussion was guided by posters for each domain of importance offering (in accessible lay language) themes, quotations from participants from previous research^19^ and questions about potential content (based on selected BCTs) (figure 1). On completion of workshops, we were confident data saturation was achieved as no new ideas were emerging.^32^

### Analysis

Workshops and individual sessions were audio recorded and transcribed. The coding framework was devised based on previously identified barriers and facilitators.^19^ Two researchers (JD and MW) coded three transcripts and discussed any differences until consistency was achieved and the remaining transcripts were then coded. Analysis was sense-checked across the author team. We did not attempt respondent validation to reduce burden on participants (who were either busy practitioners or older, often frail adults).

## Results

### Participant characteristics

There were 10 codesign participants. Table 2 illustrates the roles of participants and order of data collection.

**Table 2:** Participant characteristics codesign workshops

Pseudonym	Role	Workshop					
		1	2	3	4	5	6
Anna	Occupational therapist	✔			✔		
Meg	Physiotherapist/manager				✔		
Belle	Nurse	✔			✔		
Betty	Nurse specialist				✔		
Freda	Nurse specialist				✔		
Helga	Community matron	✔			✔		
George	Patient with PU		✔				
Cass	Carer of person with PU			✔			
Diana	Carer of husband					✔	
Patti	Carer of husband						✔

PUpressure ulcer

### Findings

Early workshops generated the most ideas for strategies to support PU prevention behaviours. These were refined in subsequent discussions and in the case of workshops 4, 5 and 6, underwent voting or prioritising activities. Strategies considered to be most acceptable, practicable and likeliest to be effective are presented below according to the barriers identified. Findings are presented according to the themes which had emerged from our previous qualitative study,^19^ which informed the content of the workshops.

#### Theme 1: knowledge and beliefs about consequences

Barriers in this theme comprised content, source and timing of knowledge and the taboo nature of PUs. Although existing information could have value, ‘*it does explain what the pressure ulcer is and the risks’* (Anna, practitioner), codesigners identified multiple limitations. Leaflets currently distributed were ‘*just another piece of information to read … just text’* (Anna, practitioner) and lacking in critical content, for example, advice about sources of practical or emotional support. Suggested improvements included using clear, understandable language, ‘*they don’t know what a pressure ulcer is. They know them as bedsores, most of them’* Helga (practitioner) and accessible text and illustrations ‘*illustrated, not* [just] *print … large so they can see it’* (Velma, carer). Messages appearing to come from a trusted or relatable person could enhance value, for example, ‘*the friendly face of a nurse’* alongside using words of real people in ‘*speech bubbles’* (Mick, patient). Views on links to videos and online resources varied, the comment ‘*not everybody would have the technology’* (Velma, carer) was countered with the suggestion ‘*get your daughter to* [help] *or something like that’* (Mick, patient).

Timing of PU prevention information provision was often suboptimal and efforts need to be made to inform people before a PU occurs. Other than existing routes to distribution (mainly via community nurses) codesigners suggested a prompt on the GP record system when a diagnosis is coded which suggests increased PU risk: ‘*a red flag should pop up, give them pressure ulcer* [information] … *diabetes … peripheral neuropathy, different conditions that mean they are higher risk of developing a pressure ulcer’* (Helga, practitioner). Other potential routes included to older people on discharge from hospital, with invitations to annual ‘influenza’ vaccinations, in ‘*the* [local] *magazine that comes out for pensioners’* (Velma, carer), posters in GPs surgeries, library, distributed by pharmacists, the Red Cross and other charities, social care practitioners (including social workers), paid carers, with the delivery of any equipment, ambulance staff and carers groups.

Other than using words and images of real people on written information, there were few suggestions about addressing taboo. The most popular suggestion was open presentations about PUs and prevention, for example, to carers groups. Velma (carer) said ‘*it’s embarrassing, people find it embarrassing, but if somebody came to talk about it, that’s a different matter because it doesn’t say you have one’*. Suggestions about television campaigns and inclusion of PU story lines in ‘*soap operas’* were quickly dismissed as being undesirable, expensive or ineffective ‘*pressure sores in* [soap opera names] … *no’* (Mick, patient).

#### Theme 2: social or professional role and influence

Considerations in this theme included uncertainty about who does what and conflicting advice and disagreements. Who does what for PU prevention was unclear for all ‘*I think we are confused as well; I don’t think it’s just the patients’* (Belle, practitioner). Patients and carers were often visited by multiple staff whose role and function was not always clear ‘*What, as a recipient of visits, I need from anybody is an introduction, good morning, I’m Angie and I’m here to do THIS. That should be an absolute mantra … often that is not the case’* (Mick, patient). Others suggested a list of names and role of staff visiting each person should be added to an information leaflet. All participants agreed that PU prevention should be a responsibility for anyone providing health or social care. To support this, codesigners agreed all staff visiting people at home should have a basic understanding of PU prevention behaviours and be able to advise patients and carers on these and know when to escalate a concern to a district nurse. Helga (practitioner) stated ‘[district nurses] *should be the last port of call when it comes to pressure prevention. If you educate everyone to the same level, so everyone has the same knowledge because I see it all the time, Fred told me to do this and Sally told me to do that and then you go in and say something different’*. Achieving fundamental and consistent knowledge for staff was considered challenging in terms of cost for providers and opportunity for staff. This was particularly so for staff employed by agencies who patients suggested had variable levels of knowledge, ‘c*are from a care agency is very sporadic, sometimes you can get five days in a row, absolutely spot on trained staff. Sometimes you get three days in a row, complete disaster. Sometimes I’ve had to tell people … this is how you put the sling in’* (Mick, patient). Although staff training was the preferred option, codesigners ultimately agreed that basic PU prevention information (with links to additional sources) on the patient leaflet (see above) could be of value to health and social carers too. It was considered this would not only address disagreements between practitioners but also support consistency within families, for example, Velma (carer) thought official information would prevent ‘*not arguments, but a disagreement’* with her husband when he thought she was ‘*keeping on’*. She said, ‘*it’s better if it comes from a nurse, rather than a person they are close to’*.

#### Theme 3: motivation and priorities

Motivation and priorities encompassed competing self-care needs and carer physical ability. Practitioners recognised conflicting needs and self-care demands experienced by patients. Pain often prevented effective repositioning and required expert nursing advice, ‘*a big factor is managing their pain before you even look at how you offload pressure’* (Helga, practitioner). Practitioners knew alternative approaches to repositioning ‘*finding different ways… there is equipment we can use … without touching … without being that intrusive’* (Anna, practitioner). Patients and their carers were not aware of these strategies which appeared to be the domain of clinicians only. Patient and carer suggestions were in relation to equipment to support repositioning, but of course, they only knew about devices they had experienced rather than the full spectrum of options. For example, Patti (carer) explained, ‘*the bed upstairs we had bits he could grab … and we’ve since had a second one on the other side … and the slider sheet’*.

#### Theme 4: emotion

Emotion centred on carer exhaustion and isolation, the challenges of carer role vs partner role and patient feelings. Carers expressed relief when quotes (see figure 1) about emotional burden were shared. Velma (carer) stated, ‘*that’s valued, those quotes … I can identify, I really can’*, similarly Diana (carer) said ‘*I don’t think people always understand how hard it is, because you have gone from a life with a husband and a wife and suddenly it isn’t like that’*. All participants saw potential benefits from support groups. One experience was recounted ‘*one of the ladies that help, you know, she came over and said* [name] *can I bring a man who is on his own to your table … of course … we became friends … we used to play cards… I would say* [name of man] *does that or he says that … talk to someone else with the same problems’* (Diana, carer). Participants acknowledged that support groups were already available locally but needed to be more effectively signposted via an information leaflet. The practicalities of getting out and about were a barrier and some suggested offering a sitting service, ‘*if they carried on the sitting service … you can go out for two hours … someone to talk to’* (Velma, carer). Staff participants were convinced the sitting service was operational and that carers were entitled to this, ‘*get your six hours of sitting service every week … it’s not means tested’.* (Meg, practitioner), however, she went on to explain ‘*it’s not any care, they might, you know gently walk, support the patient to the toilet, but it’s literally a sitting service … they are not there to provide care’*. Lack of awareness of existing services was also common among staff, this realisation brought the conversation back to written information for patients, carers and health and social care workers.

Some practitioners suggested emotional support from relatives, but recognised potential barriers to this, ‘*maybe they feel their family members don’t understand*…’ (Belle, practitioner). Carers were reluctant to burden relatives, ‘*you don’t … I didn’t worry my family … you don’t want to feel like you’re moaning all the time … when they come to see their dad, you want it to be a happy time’* (Velma, carer). Personal matters were considered unsuitable for discussion with some family members, ‘*I am very close to my son, I had a conversation with him… nitty gritty, using words like anus … it was clear from his reaction it was a one off’* (Mick, patient). However, patients and carers would ask for and receive practical support and company from their relatives.

#### Theme 5: environment

The notion of environment covered human resources and responses. Easy and effective communication was challenging to patients and staff alike with examples including frustrations around inability to contact people and changes in appointments. Codesigners suggested a dedicated telephone helpline, a service ‘*like 111’* (Belle, practitioner) where they would direct the patient or carer or pursue services, ‘*chase medicines, prescriptions, rearrange appointments’* (Anna, practitioner). Such a service may alleviate staff pressures ‘*it is down to us, a lot of the chasing, it’s taking us away from visiting the patient’* (Helga, practitioner) as well as benefiting patients and carers. Velma (carer) remembered ‘*their number was available weekends … they used to have a central number… you rang… and that person passed a message on … and the community nurse would ring and say, do you need a visit today, and it worked quite well I must say’*. Although an ideal option, consensus was that in the current UK, National Health Service climate this idea was unfeasible. In reality, many patients relied on relatives to help with this type of practicality.

Once an exhaustive list of solutions was generated, voting or prioritising exercises led to four prioritised intervention components described below with mean scores in brackets.

1. Making every contact count, all health and social care workers to be conversant with basic PU prevention behaviours and to support and reiterate these at every visit (9.1/10).
2. Signposting of existing support groups and sitting services (8.4/10).
3. Accessible, timely, trustable and relatable written and illustrated information about PUs, their causes and prevention behaviours, role of patients, carers and staff in prevention and links to other resources (7.7/10).
4. Supporting close family involvement in some of the practical elements of care (including (re) arranging appointments) (5.6/10).

## Discussion

In codesign groups involving six HCPs and four patients and lay carers, informed by data from a previous study^19^ and the TDF,^14^ participants sought to find interventions to address identified barriers in the domains of (1) knowledge and beliefs about consequences, (2) social or professional role and influence, (3) motivation and priorities, (4) emotion and (5) environment. Prioritised interventions included making ‘every contact count’ to enhance and reiterate the importance of engaging in PU prevention behaviours, signposting to support services, providing accessible written information about PU prevention but more importantly clarity about who was responsible for what and signposting links to further information and support and maximising lay help as and when appropriate.

To date, studies regarding self-management of PU prevention by community-dwelling older people and their lay carers are limited. A Cochrane review of educational interventions (n=10) reports uncertainty about the effectiveness of this approach due mainly to risk of bias and serious imprecision.^11^ Given the current understanding that theoretically underpinned interventions are more effective than those that are not,^12 13^ this is not a surprising finding.

Our target group—community-dwelling older people at risk of PUs—are likely living with one or more long-term conditions (LTCs).^33^ Self-managing any LTC requires numerous and parallel patient activities,^34^ with multiple LTCs the number of tasks to be completed can be overwhelming.^30^ A recent review reports that people living with complex LTCs are at risk of depression, psychological suffering and low self-efficacy; they often receive contradictory advice from HCPs.^35^ Patients can be faced with competing self-management demands which have to be prioritised and resourced,^36^ often they prioritise one dominant condition.^37^ They face daily iterative decisions, having to adjust their actions according to symptoms, personal priorities and available resources.^38 39^ Our codesigned prototype intervention addresses these challenges to a certain degree. It addresses the need for early PU prevention advice, perhaps a more explicit expectation of self-management and potential to reduce conflicting advice. However, we suggest skilled practitioner input is required to support patients and lay carers to address the six components of effective self-management: decision-making, action planning, partnerships with HCPs, self-tailoring, resource use and problem solving.^40^ Additionally, drawing on existing patient and lay carer self-management expertise and experience can enhance success.^35^ Therefore, further development work is needed before trialling the intervention.

Our study has both strengths and limitations. The structured theoretical approach taken increases the likelihood that selected interventions will be acceptable and practicable to end-users, and therefore, implemented in practice. Codesign was planned using best practice principles^24^ but had to be adapted due to the challenges of recruiting older and housebound people, logistics and COVID-19 pandemic restrictions. We were not able to bring HCPs and lay people together, we mitigated this by diligent iterative information sharing between and across groups. While we recruited fewer patients than carers, this is mitigated by this study building on our previous work^19^ in which 10 patients and 10 carers participated. As with all qualitative research, there is the potential for misinterpretation, however, this was mitigated through the iterative approach adopted whereby findings from previous workshops were fed into subsequent ones. This is one of few studies to consider PU prevention in community-dwelling older people. Recruitment was from one geographical area only thus potentially limiting transferability to other national and international contexts where needs and service provision may differ.

## Conclusion

Self-management and lay carer management of PU prevention is an under-researched area of risk that is increasing as the population ages and health and social care resources are ever more stretched. Our study sought to codesign with HCPs, patients and lay carers a theoretically underpinned, HCP-mediated, tailored intervention to support housebound older patients and their lay carers adopt PU prevention behaviours. The components of an intervention were developed using codesign adapted to ensure inclusivity. The process of barrier identification and selection of BCTs for intervention components was theoretically driven. However, further development will be needed to refine the prototype intervention to situate PU prevention in the context of individual’s wider health needs and priorities. The principles of this study are likely to be transferable to similar national and international contexts.

## Data Availability

Data are available on reasonable request.
