# Supplementary material for: Theoretically informed codesign of a tailored intervention to support pressure ulcer prevention behaviours by older people living in their own homes in the UK and their lay carers: an intervention codesign study (C-PrUP)
Source: BMJ Open. 2024 Nov 7;14(11):e083495. doi: 10.1136/bmjopen-2023-083495 (PMC11552011; doi:10.1136/bmjopen-2023-083495)
Supplement: Supplementary file 1 [file bmjopen-2023-083495.reviewer_comments.pdf]

PEER REVIEW HISTORY

BMJ Open publishes all reviews undertaken for accepted manuscripts. Reviewers are asked to complete a checklist review form and are provided with free text boxes to elaborate on their assessment. These free text comments are reproduced below.

ARTICLE DETAILS

Title (Provisional)

Theoretically informed co-design of a tailored intervention to support pressure ulcer prevention behaviours by older people living in their own homes in the UK and their lay carers: an intervention co-design study (C-PrUP)

Authors

Woodhouse, Marjolein; Cowdell, Fiona; Roddis, Jennifer; Devrell, Anne; Oakley, Karen; Dyson, Judith

---

VERSION 1 - REVIEW

**Reviewer**	**1**
**Name**	**Jiang, Qixia**
**Affiliation**	**Nanjing Jinling Hospital, Department of Burns and Plastic Surgery**
**Date**	**01-Feb-2024**
**COI**	**none**

This study is only a qualitative study, and the representativeness of the included objects is not sufficient. The entire article only sees the thoughts or needs of different personnel, without seeing a tailored intervention for pressure ulcer based theoretically informed co-design, which is difficult to understand.

1.On the page 4, The authors described as "We aimed to recruit between 6 and 10 HCPs and similar numbers of patients or lay carers which we judged (based on similar precedent studies27-29) to be sufficient to achieve a prototype, HCP mediated, intervention to support patients and lay carers in PU prevention behaviours. "

However, as shown in Table 2 of the results, a total of 10 cases were included in this study.

Among them, one pressure ulcer patient,Four nurses from different positions,3 caregivers.

It seems that the discrepancy between this result and the number and classification of the recruited target population. Will it affect the authenticity and reliability of the results?

On the page 10， The authors described as“Once an exhaustive list of solutions was generated, voting or prioritising exercises led to four prioritised intervention components described below with mean score in brackets.

i) Making every contact count, all health and social care workers to be conversant with basic PU prevention behaviours and to support and reiterate these at every visit (9.1/10),

ii) Signposting of existing support groups and sitting services (8.4/10),

iii) Accessible, timely, trustable and relatable written and illustrated information about PUs, their causes and prevention behaviours, role of patients, carers and staff in prevention and links to other resources (7.7/10),

iv) Supporting close family involvement in some of the practical elements of care (including (re) arranging appointments) (5.6/10).”Readers may have the following questions about this section: What is the relationship between these results and the theme of this article? What is the specific content of the intervention plan? How to demonstrate “a tailored intervention ”?

**Reviewer**	**2**
**Name**	**Holloway, Samantha**
**Affiliation**	**Cardiff University, Centre for Medical Education**
**Date**	**07-Feb-2024**
**COI**	**None**

Thank you for this interesting manuscript of an important topic. Overall the study is described well and meets the intended aim / objective. There are a few aspects of reporting that would enhance the manuscript:

Recruitment strategy - it would be useful to know when this took place i.e. which year and over how many months / which months. This would help for context. Also, did some people choose not to take part? If so why might this be?

Data collection - I would like to see mention of data saturation, even if you feel this may not apply to your methodology please explain why.

Relationship between research and participants in relation to potential bias could be addressed

Findings - a little more discussion of the credibility of the findings - triangulation, respondent validation for example

Reporting standard / checklist - was a checklist provided separately? i.e. the SRQR checklist

---

**Reviewer**	**3**

Name Palfreyman, Simon

Affiliation University of Alberta Faculty of Nursing

Date 03-Apr-2024

COI None

The manuscript and study were potentially of great interest and relevance. However, there were some areas where additional detail and explanation would have been beneficial. My comments in no particular order on the manuscript were:

1. It would have been helpful for the authors to more clearly define and justify the sampling and number of participants. The authors mention that they used judgment and similar precedents, but it is not clear how this relates to the current study.

2. The authors conflate lay carers and patients at risk of pressure ulcers. However, the sample consists of 3 lay carers and 1 person at risk. How might this impact the generalizability and interpretation of the data. The lack of patients and their insights would seem to be a major omission. Also, the patient included is currently living with a PU. Again, might this be different than someone at risk of developing a PU? What insight and differences might relying on carers impact on their results?

3. The authors refer to "pressure ulcers" throughout the manuscript. It might be useful to include other names such as pressure injuries etc.

4. More information would be useful in regard to the justification of using TDF in this particular situation.

5. Some aspects of the reporting lack precision and are vague. For example, the number of workshops and how the sample size was determined are not specified. How exactly was their approach "modified based on presidents"?

6. It would be useful to know who the carers were caring for. For example, grade, location or current pressure injury. These details would also helpful for the patient participant.

7. How was risk of PU measured/ decided?

8. How does the "earlier study" relate to the current study?

9. The were no details regarding the analysis and interpretation of the qualitative data.

10. It was unclear how the themes were arrived at and how they might have emerged from the data.

11. It was also unclear how the "exhaustive list of solutions" emerged from the data.

12. The limitations of the study need to be expanded to address issues including sample, interpretation and generalisability

VERSION 1 - AUTHOR RESPONSE

Reviewer comments	***Response:***
Reviewer 1 comments:	
This study is only a qualitative study, and the representativeness of the included objects is not sufficient. The entire article only sees the thoughts or needs of different personnel, without seeing a tailored intervention for pressure ulcer based theoretically informed co-design, which is difficult to understand.	Thank you for this insight. This is an implementation intervention (clarified on page 4 – objective). The strategies for pressure ulcer prevention are well evidenced and we report these on page 3 in our introduction. It is true this is a qualitative study, and although we do not aim for representativeness we do aim for transferability (relevance to other similar contexts). We consider the limits to transferability in our discussion on page 12.<br><br>We have added text to clarify not only the good practice guidance for co working and the theoretical underpinning (page 3) but also added our previous underpinning qualitative work (page 3 and 4) and our adoption of the MRC guidelines for complex interventions (including implementation interventions). We have added text to illustrate/add detail about our previous work on pages 5-6 (section co-design workshops) which underpinned the workshops reported here. We have also cited precedents of the approach (with similar numbers of participants – page 6) and cited seminal work about data saturation in theoretically underpinned studies such as this (page 5).
1.On the page 4, The authors described as "We aimed to recruit between 6 and 10 HCPs and similar numbers of patients or lay carers which we judged (based on similar precedent studies27-29) to be sufficient to achieve a prototype, HCP mediated, intervention to support patients and lay carers in PU prevention behaviours. "<br>However, as shown in Table 2 of the results, a total of 10 cases were included in this study.<br>Among them, one pressure ulcer patient, Four nurses from different positions,3 caregivers.<br>It seems that the discrepancy between this result and the number and classification of the recruited target population. Will it affect	Apologies, this was an incorrect statement on our part, we proposed to involve up to 7 HCPs (including a manager) and 6 patients/ lay carers. This has now been clarified (page 5 and Table 2) to demonstrate that we involved 6 HCPs, one of whom was a manager, together with 3 lay carers and a patient.<br><br>We have additionally provided clarification around the development of the intervention, which was informed by previous qualitative research (Roddis et al 2024) thus confirming authenticity of the results (page 5).

the authenticity and reliability of the results?	
On the page 10, The authors described as "Once an exhaustive list of solutions was generated, voting or prioritising exercises led to four prioritised intervention components described below with mean score in brackets.<br>i) Making every contact count, all health and social care workers to be conversant with basic PU prevention behaviours and to support and reiterate these at every visit (9.1/10),<br>ii) Signposting of existing support groups and sitting services (8.4/10),<br>iii) Accessible, timely, trustable and relatable written and illustrated information about PUs, their causes and prevention behaviours, role of patients, carers and staff in prevention and links to other resources (7.7/10),<br>iv) Supporting close family involvement in some of the practical elements of care (including (re) arranging appointments) (5.6/10)."Readers may have the following questions about this section: What is the relationship between these results and the theme of this article? What is the specific content of the intervention plan? How to demonstrate "a tailored intervention "?	Thank you for these questions, which we hope we have addressed below.<br><br>The article aims to describe the co-design of an implementation intervention to support pressure ulcer prevention behaviours. The four intervention components are the output of the co-design process (clarified on page 11).<br><br>The specific content of the intervention plan is as described on page 11. As above, the intervention consists of a series of strategies to be adopted by various individuals and does not consist solely of a physical output.<br><br>The intervention was tailored to barriers and facilitators identified in our previous work. That is – our intervention components addressed barriers and enhanced existing facilitators. We have clarified this and added a supporting reference.
Reviewer 2's comments:	
Thank you for this interesting manuscript of an important topic. Overall the study is described well and meets the intended aim / objective.	Thank you we appreciate your positive feedback and constructive suggestions to enhance the manuscript.
Recruitment strategy - it would be useful to know when this took place i.e. which year and over how many months / which months. This would help for context. Also, did some people choose not to take part? If so why might this be?	Page 5 has been updated to address these comments.
Data collection - I would like to see mention of data saturation, even if you feel this may not apply to your methodology please explain why.	We have added our guide for saturation in our background section with reference (page 5) and again our analysis section (page 6).

Relationship between research and participants in relation to potential bias could be addressed	The researchers had no existing relationship with lay participants. Some healthcare professionals were known to one researcher (MW) in a professional capacity only. There is therefore a minimal risk of bias. We have added this detail to our manuscript (page 6).
Findings - a little more discussion of the credibility of the findings - triangulation, respondent validation for example	We did not attempt respondent validation to reduce burden to participants (who were either busy practitioners or older, often frail adults). We did however ensure reliability of coding. We have written in our process for this on page 6.
Reporting standard / checklist - was a checklist provided separately? i.e. the SRQR checklist	COREQ checklist now attached
Reviewer 3's comments:	
The manuscript and study were potentially of great interest and relevance.	Thank you we appreciate your positive feedback and constructive suggestions to enhance the manuscript.
1. It would have been helpful for the authors to more clearly define and justify the sampling and number of participants. The authors mention that they used judgment and similar precedents, but it is not clear how this relates to the current study.	We agree and appreciate this feedback. We have added in justification of numbers (see responses above) on page 5 (based on theory and precedent).
2. The authors conflate lay carers and patients at risk of pressure ulcers. However, the sample consists of 3 lay carers and 1 person at risk. How might this impact the generalizability and interpretation of the data. The lack of patients and their insights would seem to be a major omission. Also, the patient included is currently living with a PU. Again, might this be different than someone at risk of developing a PU? What insight and differences might relying on carers impact on their results?	We agree this is a limitation and we have written this into our discussion section on page 12. However, the study reported here builds on our previous work where larger numbers of patients were involved. We have now written the previous work in more clearly and offered reference.
3. The authors refer to "pressure ulcers" throughout the manuscript. It might be useful to include other names such as pressure injuries etc.	Introduction amended to include alternative terms (page 3).

4. More information would be useful in regard to the justification of using TDF in this particular situation.	Unlike models of health behaviour or behaviour change, the TDF is accessible to and has been used with practitioners and service users and therefore supports the "working with" principles of co-design. We have added this explanation and supportive references to our manuscript (page 3).
5. Some aspects of the reporting lack precision and are vague. For example, the number of workshops and how the sample size was determined are not specified. How exactly was their approach "modified based on presidents"?	The number of workshops has been provided in table 2 and on page 5. Further clarification of the total number of workshops has been added on page 5.<br><br>We have clarified the modifications and cited precedents on page 5/6.
6. It would be useful to know who the carers were caring for. For example, grade, location or current pressure injury. These details would also helpful for the patient participant.	Our focus was on pressure ulcer prevention and therefore we did not routinely collect these data.
7. How was risk of PU measured/ decided?	This is now stated in the section 'Participants' (page 5).
8. How does the "earlier study" relate to the current study?	Clarification of this point is now provided on pages 5 and 7 (and as described above).
9. The were no details regarding the analysis and interpretation of the qualitative data.	Further detail has been provided (page 6).
10. It was unclear how the themes were arrived at and how they might have emerged from the data.	The themes were determined by our previous qualitative research. We agree this was unclear and we have clarified in the manuscript (pages 5 and 7).
11. It was also unclear how the "exhaustive list of solutions" emerged from the data.	Further detail about the content guiding the workshops has been provided together with further detail about data analysis are now offered on pages 5, 6 and 7 of the manuscript. We have also written in our data saturation assessment (see sections above and pages 5-7 on the manuscript).
12. The limitations of the study need to be expanded to address issues including sample, interpretation and generalisability	We have added in further limitations based on the supportive comments of the reviewers.

VERSION 2 - REVIEW

Reviewer 2

Name Holloway, Samantha

Affiliation Cardiff University, Centre for Medical Education

Date 05-Sep-2024

COI No competing interests although one of the authors - Karen Oakley is known to me as an alumnus of the Masters in Wound Healing and Tissue Repair. This was unknown to me on first review so I do not think this is a conflict.

Thank you for your revised manuscript which address my comments on the first submission.

VERSION 2 - AUTHOR RESPONSE

VERSION 3 - REVIEW

Reviewer 2

Name Holloway, Samantha

Affiliation Cardiff University, Centre for Medical Education

Date 14-Oct-2024

COI

Thank you for addressing the comments and revising the manuscript.
